# The therapeutic potential of GLP‐1 receptor biased agonism

**DOI:** 10.1111/bph.15497

**Published:** 2021-05-20

**Authors:** Ben Jones

**Affiliations:** ^1^ Section of Endocrinology and Investigative Medicine Imperial College London London UK

**Keywords:** appetite regulation, biased agonism, glucagon‐like peptide‐1 receptor, insulin release, β‐arrestin

## Abstract

Glucagon‐like peptide‐1 (GLP‐1) receptor agonists are effective treatments for type 2 diabetes as they stimulate insulin release and promote weight loss through appetite suppression. Their main side effect is nausea. All approved GLP‐1 agonists are full agonists across multiple signalling pathways. However, selective engagement with specific intracellular effectors, or biased agonism, has been touted as a means to improve GLP‐1 agonists therapeutic efficacy. In this review, I critically examine how GLP‐1 receptor‐mediated intracellular signalling is linked to physiological responses and discuss the implications of recent studies investigating the metabolic effects of biased GLP‐1 agonists. Overall, there is little conclusive evidence that beneficial and adverse effects of GLP‐1 agonists are attributable to distinct, nonoverlapping signalling pathways. Instead, G protein‐biased GLP‐1 agonists appear to achieve enhanced anti‐hyperglycaemic efficacy by avoiding GLP‐1 receptor desensitisation and downregulation, partly via reduced β‐arrestin recruitment. This effect seemingly applies more to insulin release than to appetite regulation and nausea, possible reasons for which are discussed. At present, most evidence derives from cellular and animal studies, and more human data are required to determine whether this approach represents a genuine therapeutic advance.

**LINKED ARTICLES:**

This article is part of a themed issue on GLP1 receptor ligands (BJP 75th Anniversary). To view the other articles in this section visit http://onlinelibrary.wiley.com/doi/10.1111/bph.v179.4/issuetoc

**Abbreviations**
Epac2: Exchange protein activated by cAMP‐2
GIP: gastric inhibitory polypeptide**/**glucagon‐dependent insulinotropic polypeptide

## 1 INTRODUCTION

The glucagon‐like peptide‐1 (GLP‐1) receptor is a physiologically and pharmacologically important GPCR expressed particularly in pancreatic beta cells, the brain and other metabolically relevant tissues. Its primary endogenous ligand is GLP‐1(7‐36) amide (referred to in this work as GLP‐1). The major effects of GLP‐1 include potentiation of glucose‐stimulated insulin secretion, suppression of appetite and slowing of gastric emptying (Müller et al., 2019). Five GLP‐1 mimetic agents are approved for the treatment of type 2 diabetes, including exenatide, lixisenatide, liraglutide, dulaglutide and semaglutide (Boer & Holst, 2020). GLP‐1 agonist treatment improves glycaemia, aids weight loss, improves cardiovascular, renal and limb outcomes, and leads to a reduction in all‐cause mortality in type 2 diabetes (Kristensen et al., 2019; Zheng et al., 2018). GLP‐1 agonists are also effective in causing weight loss in people without type 2 diabetes (Davies et al., 2015) and for non‐alcoholic fatty liver disease (Armstrong et al., 2016). There is emerging evidence for other beneficial actions of GLP‐1 agonists, including preservation of pancreatic beta cell mass (Boland et al., 2017), protection against neurodegenerative processes including Alzheimer's and Parkinson's disease (Grieco et al., 2019) and treatment of mood disorders (Kim et al., 2020).

A number of adverse effects of GLP‐1 agonist treatment have been reported, although not all are supported by recent evidence. Nausea is the commonest side effect, affecting 20%–50% of individuals (Bettge et al., 2016). For many, this is self‐limiting and temporary. However, some people are more severely affected and cannot continue treatment (Shiomi et al., 2019). Real‐world experience suggests the extent of the problem may not be fully represented in clinical trials (Sikirica et al., 2017). Previous concerns have been raised about the potential for GLP‐1 agonists to cause pancreatitis (Garber et al., 2009), although this has not been confirmed in recent analyses (Storgaard et al., 2017). Similarly, there was initial apprehension that GLP‐1 agonists might induce unwanted cell proliferation and increase the risk of cancer (Butler et al., 2013), but large meta‐analyses do not support this possibility (Kristensen et al., 2019). Like other GPCRs, the GLP‐1 receptor is subject to mechanisms that prevent overstimulation, which are physiologically important but may limit drug efficacy. The effect of GLP‐1 on gastric emptying undergoes rapid tachyphylaxis (Nauck et al., 2011) and this observation has been used to support the use of “short‐acting” GLP‐1 agonists that allow a between‐dose re‐sensitisation period and restoration of the beneficial effects of gastric emptying on postprandial glycaemia (Rayner et al., 2020).

Current leading GLP‐1 receptor agonist peptides were developed for prolonged pharmacokinetics compatible with once‐weekly administration in humans. Successful strategies to achieve this include amino acid sequence modifications that confer resistance to proteolytic enzymes, conjugation to fatty acids to allow reversible binding to albumin and avoidance of glomerular filtration and covalent linkage to antibody fragments for the same purpose (Andersen et al., 2018). The peptide components of all approved GLP‐1 agonists are derived from either GLP‐1 itself or its paralogue exendin‐4. Both GLP‐1 and exendin‐4 are low nanomolar affinity GLP‐1 agonists with high potency for cAMP production (de Graaf et al., 2016). Retaining these pharmacological attributes in the context of the structural modifications needed to enhance pharmacokinetics was a core tenet of the structure activity relationships strategy for liraglutide and semaglutide (Knudsen & Lau, 2019). However, greater recognition of the complexities of GPCR signalling has prompted re‐evaluation of this approach. There is increasing interest in the idea that it may be possible to produce more effective and more tolerable GLP‐1 agonists through fine tuning engagement with downstream signalling networks, a concept commonly referred to as biased agonism (Kenakin, 2018). A number of biased GLP‐1 agonists have now been reported in the literature.

In this review, I consider the following key questions:‐

1. Which proximal effectors does the activated GLP‐1 receptor primarily couple to and how are these linked to commonly measured signalling intermediates?
2. What are the physiological and therapeutic correlates of the above responses?
3. What have we learnt from recent studies testing the metabolic effects of biased GLP‐1 agonists?
4. Can beneficial and adverse effects of GLP‐1 agonists be separated according to their signalling pathway dependency?
5. What are the likely impacts of system‐ or tissue‐specific factors on biased GLP‐1 agonist responses?

This review focusses primarily on whether and how, biased GLP‐1 receptor agonism might be a means to improve treatment of type 2 diabetes and related metabolic diseases. Further details on how bias has been used to shed light on the structural basis of GLP‐1 receptor activation are not covered in detail but have been reported extensively elsewhere (Kawai et al., 2020; Liang et al., 2018; Wootten et al., 2018; Zhao et al., 2020).

## 2 MEASURING GLP‐1 RECEPTOR BIASED AGONISM *IN VITRO*

To make the pursuit of GLP‐1 receptor biased agonism therapeutically worthwhile, there should be a reason why accentuating or diminishing at least one measurable response is desirable. This condition is easily met if a therapeutic effect can be attributed to one pathway and a side effect to another. For pharmacological GLP‐1 agonists, beneficial effects include insulin secretion and weight loss and possibly additional phenomena such as beta cell and neuronal cytoprotection. Nausea is the most obvious side effect that should be avoided, although this poses the question as to whether it is possible to reduce nausea whilst still achieving effective appetite suppression and weight loss. Separately, if tachyphylaxis is a limiting factor for GLP‐1 agonist therapeutic efficacy, identifying and avoiding molecular events that underpin GLP‐1 receptor desensitisation and/or downregulation may be a fruitful approach.

Most GLP‐1 receptor biased agonist studies employ assays that can be broadly categorised as measures of either recruitment or signalling. The former includes techniques that monitor recruitment of different Gα subtypes, G‐protein receptor kinases (GRKs) or β‐arrestins to the activated GLP‐1 receptor. The latter include sensors to monitor G protein activation or, more frequently, generation of signalling intermediates including cAMP, phosphorylated ERK1/ERK2 and increases in intracellular Ca^2+^. Additionally, trafficking responses are of interest because of the potential for GLP‐1 receptor internalisation to positively or negatively modulate GLP‐1 receptor signalling by facilitating endosomal cAMP generation or, conversely, limiting the availability of surface receptors. Understanding how these readouts are linked to physiologically relevant downstream responses is of crucial importance when anticipating the pharmacodynamic profiles of novel biased GLP‐1 agonists. Moreover, some signalling assays are likely to represent the composite effect of more than one upstream process and inherent differences between how these responses are generated in different cell types could confound translation of signalling profiles from heterologous systems to native tissues. Therefore, selected components of defined GLP‐1 receptor intracellular signalling cascades are surveyed below, along with how these might be linked to physiologically or therapeutically important functions.

### 2.1 G protein selectivity of GLP‐1 receptor

Early work confirmed GLP‐1 stimulates cAMP production in islet cell lines and the brain (Drucker et al., 1987; Shimizu et al., 1987). Increases in intracellular Ca^2+^ and turnover of inositol phosphates were also noted with GLP‐1, implying a potential phospholipase C‐ (PLC‐) dependent mechanism (Wheeler et al., 1993). These findings provided preliminary evidence that GLP‐1 receptor may be capable of coupling to at least Gα_s_ and Gα_q_, which was later supported by studying G protein incorporation of [^32^P]GTP in GLP‐1 receptor‐expressing CHO cells stimulated with GLP‐1 (Montrose‐Rafizadeh et al., 1999). In the latter study, both efficacy and potency were higher for Gα_s_ than for Gα_q_, with some evidence of coupling to Gα_i_ also demonstrated. Gα_s_‐dominant coupling was also suggested by a study in which a

*Saccharomyces cerevisiae* reporter assay was used to measure GLP‐1‐induced responses (Weston et al., 2014). Here, co‐expression of GLP‐1 receptor with a yeast GPA1 protein chimera featuring mammalian Gα subtype‐specific sequences and stimulation with a high concentration (10‐μM GLP‐1), showed a response rank order of Gα_s_ > Gα_i_ > Gα_q_.

Recent studies have aimed to examine these processes via proximity‐based techniques including BRET and nano‐luciferase complementation. Using mini‐G protein probes that recognise activated GPCRs in their G protein‐favouring conformation (Wan et al., 2018), it was shown that Gα_s_ interaction with GLP‐1 receptor was significantly greater than for Gα_q_ and Gα_i_ in HEK293 cells when stimulated by GLP‐1 or exendin‐4 (Jones et al., 2020; Lucey et al., 2020). These findings are not completely definitive as mini‐G proteins are truncated and sequence‐modified Gα surrogates (Wan et al., 2018), meaning they might not faithfully replicate the interactions made by native Gα subunits. However, a high degree of selectivity for GLP‐1‐induced Gα_s_ coupling was also shown for the GLP‐1 receptor through enhanced bystander BRET to monitor translocation of full length Gα subunits to or from the plasma membrane (Avet et al., 2020). Moreover, to determine the GLP‐1 effects on G protein activation (as opposed to recruitment or redistribution) Coopman et al. (2010) measured [^35^S]GTPγS incorporation in response to GLP‐1 in HEK293‐GLP‐1 receptor membranes, demonstrating robust activation of Gα_s_ but minimal activation of Gα_q_ and Gα_i_. Recently however, measurements of heterotrimeric G protein conformational change or dissociation in response to GLP‐1 showed that GLP‐1 receptor can induce Gα_i_ as well as Gα_s_ activation, with these effects occurring over a similar ligand concentration range, albeit with different kinetics which again favour Gα_s_ as the dominant G protein response for GLP‐1 (Zhao et al., 2020). A FRET biosensor has also been used to show that 1‐nM GLP‐1 induces Gα_q_ activation in MIN6 beta cells (Oduori et al., 2020). Gα_q_ has also been implicated in GLP‐1 receptor internalisation (Thompson & Kanamarlapudi, 2015).

Overall, the available evidence favours a degree of selectivity for Gα_s_ but suggests also that GLP‐1 receptor coupling to Gα_q_ and Gα_i_ is possible under some circumstances. As tissue‐specific Gα_s_ and Gα_q_ knockout mice have been generated (Sassmann et al., 2010; Xie et al., 2007), it is unfortunate that definitive studies have not been performed to comprehensively document the *in vitro* and *in vivo* physiological responses to GLP‐1 agonists in these models. The latter study did in fact report that GLP‐1‐induced insulin secretion by isolated islets was unaffected by Gα_q_ knockout, although the concentration of GLP‐1 used was recorded as 100 μM, which would almost certainly lead to cross‐reactivity with other GPCRs. A recent study used the Gα_q_‐selective inhibitor YM‐254890 and the adenylate cyclase inhibitor MDL‐12330A to demonstrate that, under normal circumstances, GLP‐1‐induced insulin secretion is primarily Gα_s_‐mediated, but Gα_q_ plays a more important role when beta cells are chronically depolarised, for example, with sulphonylurea treatment or sustained hyperglycaemia (Oduori et al., 2020). Nevertheless, in the context of the prevailing view that GLP‐1 receptor acts predominantly via Gα_s_, concerns about the specificity of YM‐254890 (Peng et al., 2020) mean that additional independent lines of evidence are needed to verify the importance of Gα_q_ in GLP‐1 receptor signal transduction.

### 2.2 β‐arrestins—Positive or negative regulators of GLP‐1 receptor signalling?

The relative importance of canonical β‐arrestin effects, that is, termination of G protein signalling and/or initiation of GPCR endocytosis, versus the establishment of G protein‐independent signalling cascades, is a matter of ongoing debate (Grundmann et al., 2018; Luttrell et al., 2018). This is relevant to GLP‐1 receptor biased agonism as GLP‐1 receptor can interact with both β‐arrestin‐1 and β‐arrestin‐2, with similar ligand concentrations required for recruitment of either isoform (Jorgensen et al., 2007). Higher GLP‐1 concentrations are required to recruit β‐arrestins to GLP‐1 receptor than for recruitment of G proteins (Avet et al., 2020). An intramolecular BRET sensor was used to demonstrate that β‐arrestin‐2 undergoes a conformational change in response to exendin‐4 (Oishi et al., 2019) and GLP‐1 (Jones et al., 2020).

The first study to examine a putative role for β‐arrestin‐1 as a positive mediator of GLP‐1 receptor actions was from Sonoda et al. (2008), who showed reduced GLP‐1‐induced insulin secretion after β‐arrestin‐1 siRNA knockdown in INS‐1 cells. However, using mice with beta cell‐specific single knockout of β‐arrestin‐1 or β‐arrestin‐2, it has since been shown that the lack of either β‐arrestin isoform has no discernible effect on acute GLP‐1 receptor‐induced insulin secretion (Barella et al., 2019; Zhu et al., 2017). This observation matches an earlier study using global β‐arrestin‐2 knockout mice (Ravier et al., 2013). On the other hand, perifused islets from a different beta cell‐specific β‐arrestin‐1 knockout mouse model showed enhanced acute insulin secretory responses to GLP‐1 (Willard et al., 2020). Separately, it was shown that β‐arrestin‐1 may be responsible for a GLP‐1 receptor‐induced pro‐survival signal in beta cells via G protein‐independent, prolonged phosphorylation of cytoplasmic ERK1/2 (Quoyer et al., 2010). However, there was no evidence of alterations to beta cell mass in beta cell‐specific β‐arrestin‐1 or ‐2 knockout mice, albeit with the caveat that effects of chronic GLP‐1 agonist treatment were not tested in these studies (Barella et al., 2019; Zhu et al., 2017). The effects of β‐arrestin deletion on anorectic effects of GLP‐1 agonists have also not been described.

Despite the fact that GLP‐1 receptor undergoes rapid endocytosis into clathrin‐coated pits (Buenaventura et al., 2019), a phenomenon typically associated with β‐arrestin recruitment, most of the available data indicates that β‐arrestins play only a minor role in GLP‐1 receptor internalisation (Buenaventura et al., 2019; Jones et al., 2020; Jones, Bloom, et al., 2018; Thompson & Kanamarlapudi, 2015). However, prolongation of GLP‐1 receptor‐induced cAMP signalling was demonstrated in dual β‐arrestin knockout HEK293 cells (Jones et al., 2020; Jones, Bloom, et al., 2018), in line with the other well‐known action of β‐arrestins in the termination of G protein signalling, for example, through steric hindrance. Thus, β‐arrestins may well be important for desensitisation of GLP‐1 receptor‐induced G protein‐dependent signalling responses, even if not essential for GLP‐1 receptor endocytosis. However, these studies have generally been conducted in heterologous cell lines and further assessments of dynamic responses to GLP‐1 agonist treatment in β‐arrestin knockout mouse models would help clarify whether β‐arrestins serve primarily to enhance or attenuate GLP‐1 receptor signalling.

It should also be highlighted that all current approaches to delineate putative roles of β‐arrestins in the modulation of GPCR responses carry caveats. For example, individual β‐arrestin knockout mouse models may show partial compensation by the alternative isoform (Zhang et al., 2010). Moreover, cellular models with permanent deletion of β‐arrestins may lead to “rewiring” of cellular processes typically associated with β‐arrestin function (Luttrell et al., 2018). Development of small molecule inhibitors to block GLP‐1 receptor‐β‐arrestin interactions, or techniques to induce rapid and specific β‐arrestin degradation (Clift et al., 2018), would provide corroboration of results from genetic models. However, re‐routing of GPCR trafficking through alternative pathways is a recognised issue that complicates attempts to define the role of particular trafficking proteins, even with rapid‐onset pharmacological approaches (Damke et al., 1995; Dutta & Donaldson, 2012).

### 2.3 Signalling intermediates to monitor GLP‐1 receptor activation—Which pathway do they indicate?

**FIGURE 1:**
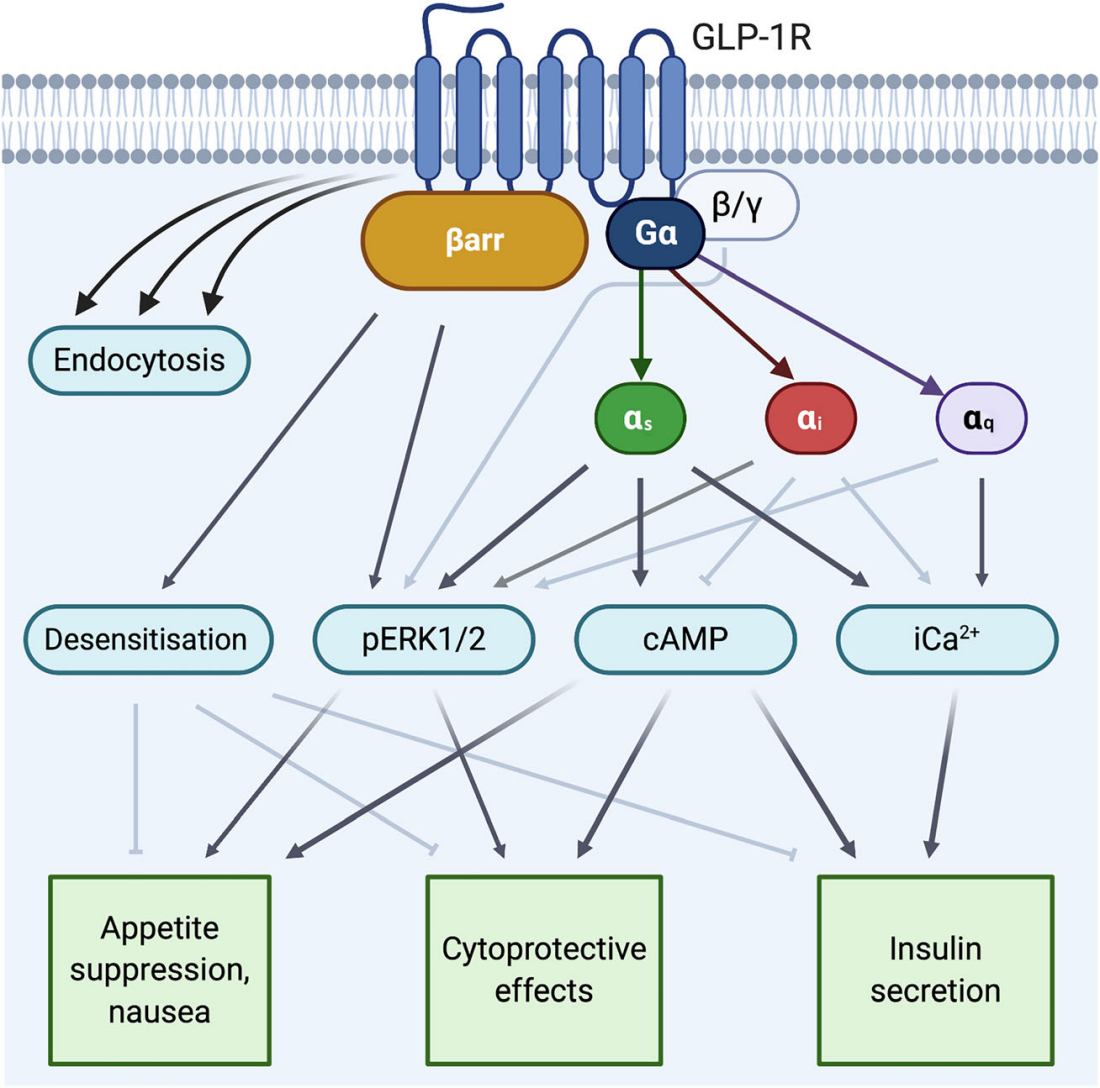
Physiological roles of different GLP‐1 receptor (GLP‐1R) signalling effectors. Commonly measured signalling intermediates may reflect the combined actions of different upstream effectors. These effects may be cell‐type specific. On balance, most evidence suggests that downstream physiological responses to GLP‐1 receptor activation are unlikely to uniquely depend on only one particular signalling pathway. Darker shaded arrows indicate that most evidence supports a link; lighter shaded arrows have some evidence base but are not fully established. Created with BioRender.com

Most current methods to monitor interactions between GLP‐1 receptor and proximal intracellular effectors, including G proteins and β‐arrestins, require overexpression of modified constructs to generate adequate signals. Recently, the feasibility of using CRISPR/Cas9 to tag endogenous proteins with the very bright luciferase NanoLuc has been demonstrated (White et al., 2017), although the required tags still might interfere with function. Biochemical measurements of signalling intermediates avoid these issues and are commonly used to monitor GLP‐1 receptor pathway activation. Whilst it is not essential that such readouts are entirely independent of each other, there is an underlying assumption that they represent distinct and meaningful responses that capture information that measurement of a single pathway does not. The likely relevance of the most widely used signalling intermediates used to profile GLP‐1 receptor activation—cAMP, intracellular Ca^2+^ and phosphorylation of ERK1/2—is therefore reviewed below, with key points summarised in Figure 1.

#### 2.3.1 cAMP

Most pharmacological studies use cAMP as a readout of GLP‐1 receptor activation. GLP‐1 receptor cAMP responses are mainly due to Gα_s_ activation, though there may be a minor cAMP‐reducing effect of Gα_i_ (Zhao et al., 2020). There is good evidence that cAMP‐dependent pathways are important for the physiological and pharmacological effects of GLP‐1. Protein kinase A (PKA), the canonical target of cAMP, was implicated as a mediator of GLP‐1‐induced insulin secretion in early studies (Fehmann et al., 1994). This has been further supported by other lines of evidence, including by observing the effects of pharmacological PKA inhibition (Skoglund et al., 2000), genetic manipulation of PKA activity (Kaihara et al., 2013) and interference with the correct localisation of PKA by disrupting A‐kinase anchoring proteins (AKAPs) (Lester et al., 1997), all of which reduce the insulinotropic action of GLP‐1. PKA signalling is also a major determinant of GLP‐1 receptor actions in different tissues, including hindbrain‐ and paraventricular nucleus‐mediated anorectic effects (Hayes et al., 2011; Liu et al., 2017), the neuroprotective effect of liraglutide in preclinical Alzheimer's disease models (Batista et al., 2018) and beneficial effects on endothelial function (Ke et al., 2017). The guanine nucleotide exchange factor Epac2 is another important cAMP‐dependent orchestrator of GLP‐1‐mediated beta cell insulin responses, as demonstrated by genetic knockdown of Epac2 in pancreatic islets (Kashima et al., 2001) and global Epac2 knockout mice (Song et al., 2013).

#### 2.3.2 Intracellular Ca^2+^

Elevation of intracellular Ca^2+^ concentrations is a late event that leads to exocytosis of insulin granules from beta cells. Measurements of intracellular Ca^2+^ have also frequently been used to assess GLP‐1 receptor biased agonism in heterologous cell systems. This begs two questions: (1) does GLP‐1 receptor increase Ca^2+^ through predominantly Gα_s_ or Gα_q_‐mediated mechanism(s) and (2) do the same mechanisms apply in both heterologous cell systems and native cell types such as beta cells and neurons?

There is abundant evidence that, in beta cells, Ca^2+^ responses are at least partly dependent on cAMP. Administration of the adenylate cyclase activator forskolin leads to increases in Ca^2+^ (Fridolf & Ahrén, 1993). PKA phosphorylates multiple targets involved in intracellular Ca^2+^ homeostasis, such as K_ATP_ channels that control membrane depolarisation (Gromada et al., 1997) and inositol triphosphate receptors that promote release of Ca^2+^ from intracellular stores (Tsuboi et al., 2003). Epac2 is also involved in K_ATP_ channel closure (Kang et al., 2008), activation of PLC‐*ɛ* (Dzhura et al., 2010) and activation of ryanodine receptors (Tsuboi et al., 2003), all of which lead to Ca^2+^ elevations. However, the possibility that GLP‐1 receptor may induce Gα_q_‐dependent Ca^2+^ release was raised by demonstrating increased inositol phosphate turnover in beta cells in response to GLP‐1 (Wheeler et al., 1993), although a number of other studies did not reach the same conclusion (Fridolf & Ahrén, 1991; Göke et al., 1989; Röthe et al., 2020; Zawalich et al., 1993). In favour of a role for Gα_q_, Shigeto et al. (2015) showed that very low concentrations of GLP‐1 (1 pM) result in the generation of diacylglycerol, interpreted to be secondary to Gα_q_‐mediated activation of PLC; it should be noted however that cAMP can also activate PLC (Liu & Simon, 1996). A panel of small molecule inhibitors was used to assess the G protein subtype dependence of GLP‐1 receptor‐induced Ca^2+^ responses in CHO‐K1 cells (Wootten et al., 2016), providing useful information on the meaning of this response in heterologous systems. Here, inhibitors of adenylate cyclase, PKA, Gβγ, Gα_q_ and Gα_i_ all reduced Ca^2+^ responses when cells were treated with pEC_50_ concentrations of GLP‐1, exendin‐4 or oxyntomodulin. The largest effect was seen with the Gα_q_ inhibitor UBO‐QIC, suggesting an important role for Gα_q_. The relative contributions of each G protein subtype cannot be unequivocally determined though as the degree of inhibition obtained is unknown, and UBO‐QIC and other inhibitors are unlikely to show absolute specificity (Gao & Jacobson, 2016).

The available data therefore suggests that GLP‐1 receptor‐induced Ca^2+^ responses represent a composite readout of activation of Gα_s_ in particular, Gα_q_ to a variable degree and possibly other G protein subunits. However, whilst intracellular Ca^2+^ is undoubtedly a physiologically important response in electrically excitable cells such as beta cells and neurons, it is unclear how well this is replicated by measuring responses in nonexcitable cells, due to cell‐specific differences in the mechanisms underpinning Ca^2+^ dynamics. For example, there are clear differences in the kinetics of Ca^2+^ elevations in CHO cells versus intact islets (Hodson et al., 2013; Wootten et al., 2011). Moreover, EC_50_ values for intracellular Ca^2+^ responses in CHO cells are right‐shifted by two orders of magnitude compared to cAMP (Koole et al., 2010) and Ca^2+^ and cAMP responses are usually measured over different time intervals—an important consideration due to the effect of kinetics on the interpretation of apparent biased agonism (Klein Herenbrink et al., 2016).

#### 2.3.3 ERK1/2 phosphorylation

Phosphorylation of ERK1/2 was one of the first GPCR responses to be highlighted as an example of β‐arrestin‐dependent, G protein‐independent signalling (Luttrell et al., 2001) and the availability of high throughput assays to measure ERK1/2 phosphorylation render it an appealing and straightforward readout that could provide information on β‐arrestin recruitment. Indeed, β‐arrestin‐1 knockdown attenuated GLP‐1‐induced ERK1/2 phosphorylation in INS‐1 cells (Sonoda et al., 2008), although other studies showed ERK1/2 phosphorylation in response to GLP‐1 is cAMP/PKA dependent (Briaud et al., 2003). These discrepancies might be rationalised by the finding that the acute phase of GLP‐1 receptor‐induced ERK1/2 phosphorylation depends on PKA but a late, cytoplasmic phase requires β‐arrestin‐1 (Quoyer et al., 2010). Matching this observation, sustained (but not acute) ERK1/2 phosphorylation was reduced in HEK293 cells lacking both β‐arrestin isoforms (Jones et al., 2020). By comparing signalling responses in CHO cells in the presence of different pharmacological inhibitors or β‐arrestin‐1/2 dominant negative isoforms, the ability of GLP‐1 receptor to induce ERK1/2 phosphorylation was concluded to depend particularly on β‐arrestin‐1, β‐arrestin‐2, Gβγ and Gα_i_, and to a lesser extent on Gα_s_ and Gα_q_ (Wootten et al., 2016).

ERK1/2 and related MAPKs are well known for their roles in cell survival, differentiation and growth. Sustained ERK1/2 phosphorylation was found be important for GLP‐1‐induced protection of beta cells against apoptosis (Quoyer et al., 2010). Pharmacological inhibition of ERK1/2 also abrogated the effects of GLP‐1 agonists on neuronal survival (Zhu et al., 2016), adipocyte differentiation (Challa et al., 2012) and osteogenic precursor proliferation (Wu et al., 2017). However, in other studies, ERK1/2 did not contribute to the cytoprotective effect of exendin‐4 (Campbell et al., 2016; Kawasaki et al., 2010) and signal bias favouring ERK1/2 with oxyntomodulin was in fact associated with bias away from anti‐apoptotic effect and proliferation in INS‐1 cells (Wootten et al., 2016). It also appears unlikely that GLP‐1 receptor‐mediated effects on cell survival via ERK1/2 signalling can be clearly separated from activation of cAMP/PKA pathways, not least as ERK1/2 phosphorylation is partly PKA dependent. The ability of exendin‐4 to protect beta cells against endoplasmic reticulum stress was shown to involve PKA (Yusta et al., 2006), with evidence also for a role for PKA in exendin‐4‐induced neuronal protection (Wang et al., 2012). Moreover, ERK1/2 was found to be involved in other GLP‐1 receptor actions including acute insulin secretion and appetite regulation (Hayes et al., 2011; Jacobo et al., 2009), suggesting there is significant overlap between the signalling pathways regulating different GLP‐1 receptor effects.

Therefore, GLP‐1 receptor‐induced ERK1/2 phosphorylation appears to be a composite activation marker for many upstream effectors, including different G protein subtypes as well as β‐arrestin recruitment. An active role for β‐arrestin in promoting ERK phosphorylation distinguishes it from G protein‐mediated cAMP and Ca^2+^ responses, at least in CHO cells, although it is not an ideal surrogate for β‐arrestin recruitment because the relative contributions of each upstream effector are ligand‐dependent (Wootten et al., 2016). There is also insufficient data to link ERK1/2 phosphorylation to a distinct GLP‐1 receptor physiological effect that is not captured by other readouts.

### 2.4 GLP‐1 receptor internalisation and endosomal signalling

GLP‐1 receptor undergoes rapid agonist‐mediated internalisation (Widmann et al., 1995). Recognition that active GPCRs can continue to generate cAMP signals from endosomal compartments (Calebiro et al., 2009) has led to increased interest in GPCR trafficking, including for the GLP‐1 receptor. Inhibition of GLP‐1 receptor internalisation using a dynamin inhibitor or hypertonic sucrose reduced exendin‐4‐induced cAMP production (Kuna et al., 2013), as did expression of a dominant negative dynamin isoform (Roed et al., 2015). Recruitment of Gα_s_ to GLP‐1 receptor‐containing endosomes has also been demonstrated (Girada et al., 2017) and a recent publication describes how mini‐G protein recruitment to different endosomal compartments with a panel of mono‐ and dual GLP‐1/GIP receptor agonists is linked to agonist‐specific receptor trafficking profiles (Novikoff et al., 2021). As well as cAMP production, dynamin ablation reduces ERK phosphorylation (Fletcher et al., 2018) and a strategy to prolong endosomal residence of exendin‐4 by covalent linkage to the SNAP‐GLP‐1 receptor led to prolongation of intracellular Ca^2+^ responses in MIN6B1 beta cells (Podewin et al., 2018). These observations suggest that full amplitude GLP‐1 receptor responses probably require the receptor to enter the endocytic pathway. However, it is also evident that activated GLP‐1 receptors are rapidly targeted to lysosomes and degraded (Jones, Bloom, et al., 2018), which may limit the availability of surface receptors during prolonged treatment. The potential for GLP‐1 receptor endocytosis to both positively and negatively regulate GLP‐1 receptor signalling may be time dependent, with different implications for short‐lived endogenous ligands that are rapidly destroyed in the circulation versus long‐lasting pharmacological agonists which persist for many days. Spatiotemporal aspects of GLP‐1 receptor signalling have been more extensively reviewed elsewhere (Manchanda et al., 2021).

## 3 BIASED GLP‐1 AGONISTS AND THEIR PHYSIOLOGICAL EFFECTS

A number of biased GLP‐1 agonists have now been described. These range from endogenous preproglucagon‐derived peptides to synthetic peptides or peptidomimetics designed for therapeutic efficacy, to small molecule GLP‐1 agonists that may be suitable for oral administration.

### 3.1 Endogenous and approved therapeutic GLP‐1 agonists

The first report to explicitly consider the possibility of biased GLP‐1 receptor agonism was by Jorgensen et al. (2007), who showed that oxyntomodulin, glucagon and exendin‐4 were full agonists for GLP‐1 receptor‐induced cAMP production but showed modestly reduced efficacy for recruitment of β‐arrestin‐1 and β‐arrestin‐2 compared to GLP‐1. However, their analysis ignored relative potency differences and formal bias quantification using standardised methodology in fact suggests that both exendin‐4 and oxyntomodulin are biased in favour of β‐arrestin‐2 recruitment and ERK1/2 phosphorylation over cAMP production, relative to GLP‐1 (Fletcher et al., 2018; Koole et al., 2010; Wootten et al., 2016). The impact of potency differences on bias can be under appreciated when concentration responses are visually inspected, due to the logarithmic concentration scale. However, other studies show at least a trend for exendin‐4 to be subtly biased in favour of cAMP production over β‐arrestin‐2 recruitment (Fremaux et al., 2019) or report relative potencies ratios in keeping with a degree of selectivity for cAMP production (Plisson et al., 2016). In yeast assays, oxyntomodulin showed enhanced coupling to Gα_i_ compared to GLP‐1 (Weston et al., 2014) which, given the possible dependence of oxyntomodulin‐induced ERK/1/2 phosphorylation on Gα_i_ (Wootten et al., 2016), could be relevant to the relative preference of this peptide for ERK1/2 signalling. The physiological implications of the differential signalling profiles between GLP‐1 and oxyntomodulin are difficult to examine *in vivo* as oxyntomodulin cross reacts with the glucagon receptor and both peptides have short circulatory half‐lives. Similarly, elucidating the impact of bias differences between GLP‐1 and exendin‐4 *in vivo* would be challenging due to their quite different pharmacokinetics.

Currently approved GLP‐1 agonists, including liraglutide, semaglutide, dulaglutide and lixisenatide, as well as exendin‐4 as mentioned above, have also been examined for evidence of bias between cAMP and β‐arrestin‐2 recruitment (Jones, Bloom, et al., 2018). All were full agonists for both pathways except exendin‐4, which showed a subtle reduction in efficacy for β‐arrestin‐2. With exendin‐4 as the comparator, only liraglutide showed statistically significant bias, favouring β‐arrestin‐2 recruitment over cAMP. However, in another study, liraglutide showed bias away from β‐arrestin‐2 compared to exendin‐4 but did favour ERK1/2 phosphorylation (Fletcher et al., 2018). Liraglutide was associated with relatively reduced coupling of GLP‐1 receptor to Gα_i_ in yeast when compared with exendin‐4 (Weston et al., 2014). Lixisenatide was also biased relative to exendin‐4 in favour of GLP‐1 receptor endocytosis over cAMP (Pickford et al., 2020). In most cases, *in vivo* comparisons of different approved GLP‐1 agonists are not straightforward due to confounding by pharmacokinetic differences.

Overall, for GLP‐1 agonists that have been independently evaluated by different research groups, such as GLP‐1 versus exendin‐4, there remains some divergence between bias estimates in the published literature. This issue, which is a significant problem affecting the biased agonism field in general (Onaran et al., 2017), could be due to a wide range of experimental factors such as use of receptor tags, host cell type, stimulation times,and assay technologies. However, most studies have shown that the aforementioned ligands are either full agonists or show only slightly reduced maximal responses for all pathways, which is not the case for more recently described biased GLP‐1 agonists (see below).

### 3.2 Novel biased GLP‐1 agonists described in the preclinical literature

The first bespoke biased GLP‐1 agonist to be reported, P5, was obtained through an innovative strategy involving expression of single peptide sequences from an exendin‐4‐derived combinatorial peptide library, with peptides anchored to the cell surface via a flexible linker that allows autocrine activation of GLP‐1 receptors expressed in the same cell (Zhang et al., 2015). This approach led to the identification of P5, in which the first 8 amino acids of exendin‐4 (HGEGTFTS) are replaced by a novel 9 amino acid sequence (ELVDNAVGG). P5 showed full agonist activity for cAMP, with a modest reduction in potency compared to exendin‐4, along with more dramatic reductions in potency and efficacy for β‐arrestin‐1 and β‐arrestin‐2 recruitment. Label‐free cellular impedance measurements suggested that P5 causes less GLP‐1 receptor desensitisation than exendin‐4. P5 was compared against exendin‐4 in a variety of mouse models of diabetes, showing an unusual pharmacodynamic profile including greater acute anti‐hyperglycaemic efficacy than exendin‐4 but reduced insulin secretion and unaffected insulin sensitivity. P5 also outperformed exendin‐4 for reducing HbA1c in a chronic administration study. The mechanism underpinning the greater glucose‐lowering effect with P5 was not fully explained, although other P5‐specific metabolic changes included increased adipocyte hyperplasia, decreased adipose tissue inflammation and increased circulating concentrations of the incretin hormone glucose‐dependent insulinotropic polypeptide/gastric inhibitory polypeptide (GIP). A recent study has demonstrated the evolution of P5, with the latest version featuring a P5‐homologous N‐terminus (ELVDCAV) fused to a modified version of GLP‐1(9–37), along with a fatty acid chain to improve pharmacokinetics (Wang et al., 2020). This peptide (PX17) was more effective than semaglutide for blood glucose lowering and weight loss when administered chronically to mice. However, whilst PX17 was described as a biased GLP‐1 agonist by the authors, this was not formally assessed in this study. As with P5, an increase in circulating GIP was seen with PX17 but not semaglutide.

In another study, a panel of exendin‐4 analogues featuring single amino acid substitutions at or close to the peptide N‐terminus was assessed for bias between cAMP production, β‐arrestin‐2 recruitment, and GLP‐1 receptor endocytosis (Jones, Bloom, et al., 2018). The peptide with the most distinctive profile, exendin‐phe1 (in which the N‐terminal histidine was switched to phenylalanine), showed markedly reduced efficacy for β‐arrestin recruitment and GLP‐1 receptor endocytosis, whilst acting as a full agonist for cAMP with modestly reduced potency. Beta cells secreted more insulin during prolonged exposure to exendin‐phe1 versus exendin‐4, with further studies highlighting fast dissociation kinetics, fast GLP‐1 receptor recycling and avoidance of GLP‐1 receptor desensitisation and degradation as key components of its mode of action. Exendin‐phe1 had a progressively larger effect on blood glucose lowering 4 and 8 h after dosing compared to exendin‐4 despite identical pharmacokinetics, suggesting the effects of minimised GLP‐1 receptor desensitisation demonstrated *in vitro* are also seen *in vivo*. Contrasting with its enhanced effects on blood glucose modulation, exendin‐phe1 led to a similar degree of appetite suppression as exendin‐4 and less pica behaviour (a mouse correlate of nausea). Effects on GIP levels were not reported, so it is unclear whether the increase in circulating GIP seen with P5 and PX17 indicate a general phenomenon with biased GLP‐1 agonists.

An acylated version of exendin‐phe1 has also been tested, which showed a directionally similar but more extreme signalling profile compared to the nonacylated form, characterised by virtually undetectable β‐arrestin recruitment. Acylated exendin‐phe1 significantly outperformed a comparator acylated peptide showing the opposite bias characteristics (acylated exendin‐asp3) for sustained blood glucose lowering (Lucey et al., 2020). Importantly, this study also showed that both acylated and nonacylated exendin‐phe1 in fact show considerably reduced efficacy for Gα_s_ engagement, as assessed by mini‐G_s_ recruitment assays. This observation highlights how partial agonists for this proximal part of the signalling pathway can still generate full amplitude cAMP signals due to the amplifying effects of adenylate cyclase. Alternatively, ligand‐specific differences in the ability of agonist‐occupied receptors to activate recruited G proteins mean that measurements of recruitment may overestimate efficacy differences. Further studies have revealed that the degree of bias conferred by the phe1 substitution is influenced by other agonist structural features, with the largest effect seen in peptides with greatest sequence homology to exendin‐4 versus GLP‐1 (Fang, Chen, Manchanda, et al., 2020; Fang, Chen, Pickford, et al., 2020). Effects on sustained insulin secretion were correlated with the degree of bias. Notably, the N‐terminal sequence phe1‐gly2, as opposed to phe1‐ala2, was identified as being required for the most dramatic reductions in β‐arrestin‐2 recruitment efficacy. The phe1 substitution was also introduced into lixisenatide and showed similar effects (Pickford et al., 2020), as was also the case when the position 2 glycine in modified forms of exendin‐4 and lixisenatide were substituted to a “ureido” oliguria residue (Fremaux et al., 2019). Recently, GLP‐1 with a valine substitution at position 2 was described as a cAMP‐biased agonist that results shows impaired acute secretion of pancreatic hormones, including insulin and somatostatin; effects on sustained release of insulin or other hormones were not described (van der Velden et al., 2021). N‐terminal modification of orthosteric GLP‐1 agonists therefore appears to be a useful way to induce G protein‐directed biased agonism.

A variety of further chemical approaches have also been used to generate biased GLP‐1 agonists. Introducing targeted amino acid substitutions to the GLP‐1 sequence with the aim of increasing helical stability resulted in enhanced cAMP signalling and insulin release, with a lesser effect on β‐arrestin‐2 recruitment which, although bias was not formally quantified in this study, again suggested that cAMP‐favouring GLP‐1 receptor agonism is preferred for insulin release (Plisson et al., 2016). In contrast, substituting GLP‐1 mid‐helical residues from α to nonnatural β‐amino acids led in some cases to bias favouring β‐arrestin recruitment (Hager et al., 2016). Notably, in the latter study, many of the β‐arrestin‐biased agonists in fact showed reduced efficacy for β‐arrestin recruitment, but high potency. Further examples of this chemical series have since been described (Cary et al., 2019; Hager et al., 2017), but it has not been reported whether the demonstrated bias profiles have an impact on insulin release or other physiological effects. Another GLP‐1 derived peptide has been described that features O‐Glc‐NAc glycosylation of ser18, as well as 2‐aminoisobutyric acid substitutions at positions 2 and 16, is biased in favour of cAMP signalling over β‐arrestin recruitment (Levine et al., 2019). This peptide showed improved anti‐hyperglycaemic efficacy versus GLP‐1, although large differences in proteolytic stability undoubtedly contribute to its *in vivo* actions.

A panel of previously described dual agonist peptides showing activity at both GLP‐1 and glucagon receptors, two in the late stages of clinical testing (MEDI0382 and SAR425899), has recently been evaluated for signal bias (Darbalaei et al., 2020). MEDI0382 (cotadutide) (Ambery et al., 2018) showed a subtle degree of bias in favour of β‐arrestin‐2 over cAMP production at the GLP‐1 receptor, as did SAR425899 (Tillner et al., 2019), although not statistically significant for the latter. Data for β‐arrestin recruitment to the glucagon receptor were not available for these peptides, so the matching glucagon receptor bias profile could not be determined; however, both showed a significant degree of bias favouring ERK phosphorylation over cAMP. What role biased signalling plays in mediating the physiological effects of these dual agonist ligands is not clear.

Recently, an orally available small molecule GLP‐1 receptor activator (LY3502970 or OWL833) was reported that shows undetectable β‐arrestin recruitment at the human GLP‐1 receptor whilst retaining the ability to stimulate cAMP production (Kawai et al., 2020). LY3502970 shows a unique mode of binding dependent on the human‐ and primate‐specific GLP‐1 receptor residue trp33, which is not the case for peptide GLP‐1 agonists. Controlling GLP‐1 receptor expression levels to modulate the availability of spare receptors showed that LY3502970 acts as a full agonist for cAMP at high receptor densities but is a partial agonist when GLP‐1 receptor expression is a limiting factor. The pharmacological characteristics of LY3502970 are reminiscent of TT‐OAD2, another nonpeptide GLP‐1 receptor agonist that also shows a high degree of selectivity for G protein signalling (Zhao et al., 2020). A newer iteration of TT‐OAD2, termed TTP273, is in clinical trials, and early, unpublished results suggest it is highly effective for blood glucose control (https://vtvtherapeutics.com/pipeline/ttp273/). Another oral small molecule GLP‐1 receptor agonist (PF‐06882961) has also been recently described which shares a similar mode of GLP‐1 receptor binding with LY3502970 but, interestingly, shows only a minor degree of bias in favour of cAMP over β‐arrestin recruitment (Griffith et al., 2020; Zhang et al., 2020).

Selected GLP‐1 agonists for which biased agonism is an important or intentional feature of their pharmacology are summarised in Table 1.

**TABLE 1:** Selected GLP‐1 receptor agonists for which biased agonism is a core or intentional feature of their pharmacology

Agonist(s)	Structure	Bias	Notable physiological effects compared to “nonbiased” agonist	Reference
P5	Peptide	cAMP > β‐arrestin	↑Anti‐hyperglycaemia, ↓acute insulin, ↑[GIP]	(Zhang et al., 2015)
PX17	Peptide	Not reported, presumed to be cAMP > β‐arrestin	↑Anti‐hyperglycaemia, ↑weight loss, ↑[GIP]	(Wang et al., 2020)
Exendin‐phe1	Peptide	cAMP > β‐arrestin and GLP‐1 receptor endocytosis	↑Anti‐hyperglycaemia, ↑sustained insulin, ↓pica	(Jones, Bloom, et al., 2018)
Acylated exendin‐phe1	Peptide	cAMP > β‐arrestin	↑Anti‐hyperglycaemia, ↑weight loss	(Lucey et al., 2020)
Ex4^L^[A^u^]^2^ (peptide 11)	Peptide	cAMP > β‐arrestin	↑Anti‐hyperglycaemia, ↑sustained insulin	(Fremaux et al., 2019)
GLP‐1‐Val8	Peptide	cAMP > β‐arrestin	↓Acute insulin and somatostatin	(van der Velden et al., 2021)
β‐Amino acid peptidomimetics	Peptide	Varies	Not described	(Hager et al., 2016; 2017; Cary et al., 2019)
O‐GlcNAc‐modified peptides	Peptide	cAMP > β‐arrestin	↑Anti‐hyperglycaemia?^a^	(Levine et al., 2019)
Tirzepatide	Peptide	cAMP > β‐arrestin	↑Anti‐hyperglycaemia, ↑weight loss^b^	(Novikoff et al., 2021; Willard et al., 2020; Yuliantie et al., 2020)
LY3502970/OWL833	Small molecule	cAMP > β‐arrestin	Orally available^c^	(Kawai et al., 2020)
TT‐OAD2	Small molecule	cAMP > β‐arrestin	Orally available^c^	(Zhao et al., 2020)
PF‐06882961	Small molecule	cAMP > β‐arrestin (mild)	Orally available^c^	(Griffith et al., 2020)

^a^ PK differences are not accounted for.

^b^ GIP receptor activity may contribute.

^c^ No suitable “non‐biased” comparator available for physiological comparisons.

### 3.3 Tirzepatide—A “twincretin” with biased GLP‐1 receptor action and promising anti‐diabetic effects in humans

Tirzepatide (LY3298176) is a dual agonist in the late stages of clinical development that acts jointly at GLP‐1 and GIP receptors, a so‐called “twincretin” (Frias et al., 2018). Promising results for tirzepatide are already described, including outperforming dulaglutide for improvements in both HbA1c and weight loss in people with type 2 diabetes. Initial descriptions of tirzepatide showed similar GIPR potency to native GIP but fivefold reduced potency for GLP‐1 receptor (Coskun et al., 2018). Even though tirzepatide is a potent activator of GIP receptor, the contribution of GIP receptor to its overall metabolic effects is not fully understood, although effects on adipose tissue are probably important (Samms et al., 2020). It has since transpired that tirzepatide is a biased GLP‐1 agonist with very low efficacy for recruitment of β‐arrestin, whilst acting as an unbiased GIP agonist with full efficacy for β‐arrestin recruitment (Willard et al., 2020; Yuliantie et al., 2020). Tirzepatide features a tyrosine at its N‐terminus, which is typically required for high GIP receptor affinity but was also shown previously to attenuate GLP‐1 receptor β‐arrestin recruitment efficacy as part of an exploration of exendin‐4 N‐terminal substitutions (Jones, Bloom, et al., 2018). Tirzepatide also shows a much‐reduced GLP‐1 receptor internalisation response. Beta cell‐specific knockout of β‐arrestin‐1 increased insulin secretion in response to GLP‐1, but not to tirzepatide, suggesting that β‐arrestin recruitment may restrain the insulinotropic response of GLP‐1 receptor activation; these findings are not entirely congruent with other studies using different beta cell‐specific β‐arrestin knockout mice however (Barella et al., 2019; Zhu et al., 2017). Further studies are needed to establish whether biased GLP‐1 receptor agonism contributes substantially to the metabolic effects of tirzepatide.

## 4 DO BIASED GLP‐1 AGONISTS SHOW DISTINCT PHYSIOLOGICAL EFFECTS?

The accumulated knowledge of how GLP‐1 receptor signalling is linked to actions *in vivo*, combined with growing experience of biased GLP‐1 agonists in preclinical disease models and humans, provides some clues as to whether biased agonism is a viable strategy to improve GLP‐1 receptor targeting in type 2 diabetes and other metabolic diseases. Semaglutide, the most effective GLP‐1 agonist currently available, has powerful effects on blood glucose and weight loss in its once‐weekly injectable formulation. An oral version is also available that is more convenient for many patients (although slightly less effective) (Nauck et al., 2020). Biased GLP‐1 agonists would need to provide distinct benefits over semaglutide to warrant the investment required to bring a new agent to market. Theoretically, a unique and beneficial effect seen only with biased GLP‐1 agonists and not with existing GLP‐1 agonists would be one potential advantage, but there is scant evidence that this will be the case. One possible example would be the unexplained effects of P5 and PX17 on circulating biomarkers including GIP and anti‐inflammatory cytokines, that were not seen with the unbiased comparator peptides (Wang et al., 2020; Zhang et al., 2015). It is more likely however that biased GLP‐1 agonists will display the usual pattern of GLP‐1 receptor metabolic actions seen with balanced GLP‐1 agonists,. The question is whether it will be possible to accentuate or minimise some of these to improve either overall anti‐diabetic efficacy or the therapeutic window.

The main adverse effect of GLP‐1 agonist treatment is nausea. Investigation of nausea is challenging in mice due to the lack of an emesis response but can be performed using conditioned taste aversion or behavioural assays (Jones, Bloom, et al., 2018) or in other rodent species such as shrews (Borner et al., 2020). Monitoring acute reductions in food intake may be an approximate surrogate of nausea in mice, although the lack of an association between gastro‐intestinal adverse effects and weight loss in GLP‐1 agonist clinical trials suggest different mechanisms may be involved in humans (Lingvay et al., 2020). Despite their markedly greater effects on blood glucose lowering, neither exendin‐phe1 nor P5 showed any increases in anorectic efficacy compared to exendin‐4 (Jones, Bloom, et al., 2018; Zhang et al., 2015). This was also the case when phe1‐substituted lixisenatide was compared against oppositely biased lixisenatide‐asp3 (Pickford et al., 2020). This does not mean biased GLP‐1 agonists are necessarily less effective for weight loss than GLP‐1 agonists with a balanced signalling profile; acylated exendin‐phe1 and PX17 achieved at least equivalent weight loss to their balanced agonist comparator peptides (Lucey et al., 2020; Wang et al., 2020). Moreover, tirzepatide outperformed dulaglutide for weight loss in type 2 diabetes (Frias et al., 2018), although it is difficult to ascribe this to biased GLP‐1 receptor agonism due to the potential for GIPR‐induced appetite suppression (Adriaenssens et al., 2019) and the fact that the large molecular size of dulaglutide may limit access to appetite regulatory centres to a greater extent than with tirzepatide. There is very limited data concerning nausea with biased GLP‐1 agonists in preclinical models, with reduced pica in mice with exendin‐phe1 versus exendin‐4 being one published example (Jones, Bloom, et al., 2018). Whilst a proper head‐to‐head comparison is required, there was a slightly lower rate of nausea (40% vs. 50%) observed with the highest dose tested of tirzepatide compared to the highest dose tested of semaglutide in separate clinical trials designed to determine the optimum dose escalation schedule for each agonist (Frias et al., 2020; O'Neil et al., 2018). Interestingly, unpublished results for the orally available biased agonist TTP273 (https://vtvtherapeutics.com/pipeline/ttp273/) show a very effective reduction in HbA1c at 12 weeks that approaches that reported separately (Frias et al., 2020) for tirzepatide (1.7% versus 2.0%), a slightly less impressive effect on weight loss (3.8 kg versus 5.5 kg) and a markedly lower incidence of nausea. Phase 1 clinical data for the modestly biased PF‐06882961, currently reported in a preprint (Griffith et al., 2020), show a small blood glucose lowering effect after a single dose, along with a relatively high rate of nausea. PF‐06882961 is currently in phase 2 clinical development, with preliminary results for type 2 diabetes patients reported in abstract form and indicating effective blood glucose lowering effect and weight loss (https://diabetes.diabetesjournals.org/content/69/Supplement_1/353-OR). Nausea rates were not reported in the online abstract.

Overall, these data do tentatively support the possibility that biased GLP‐1 agonists might be associated with a lower rate of nausea than standard agents, although GLP‐1 agonists displaying a more extreme level of bias (e.g., TTP273) might be less effective for weight loss. Of note, whilst G protein‐independent β‐arrestin‐mediated GLP‐1 receptor signalling was linked to improvements in beta cell survival (Quoyer et al., 2010), beta cell mass expansion in mice treated with acylated exendin‐phe1 was similar to with the comparator peptide showing a full β‐arrestin recruitment response (Lucey et al., 2020).

### 4.1 Is there a specific intracellular signalling pathway that mediates GLP‐1 agonist‐induced nausea?

The promise of biased agonism rests on the idea that drug side effects might derive from signalling pathways that are at least partly distinct from those responsible for therapeutic effects. A well‐known example is the μ opioid receptor, for which studies in β‐arrestin knockout mice have inspired a longstanding paradigm that analgesic effects of opioid medications are G protein‐mediated but respiratory depression depends on specific β‐arrestin‐dependent signalling (Raehal et al., 2005; Schmid et al., 2017), although this has recently been challenged through re‐evaluation of the bias profiles of purportedly biased μ agonists (Gillis, Gondin, et al., 2020; Gillis, Kliewer, et al., 2020) and failure to replicate the original findings from β‐arrestin‐2 knockout mice that supported this concept (Kliewer et al., 2020). Unfortunately, there is virtually no evidence that pinpoints particular intracellular signalling pathways as being responsible for GLP‐1 agonist‐induced nausea. These are currently no reports comparing the effects of GLP‐1 agonists on nausea or appetite suppression in β‐arrestin knockout mice or indeed in mice with selective G protein deletion. The signalling pathways underpinning neural GLP‐1 receptor actions on feeding behaviours are less well studied than those for insulin secretion by beta cells, although both PKA and ERK1/2 signalling may be involved (Hayes et al., 2011; Liu et al., 2017). Overall, it is unlikely that there exists an intracellular signalling pathway that is both highly specific for nausea and also accessible to exclusive modulation by biased GLP‐1 agonists.

### 4.2 Tissue‐specific actions of biased GLP‐1 agonists

A further possibility explaining why biased GLP‐1 agonists may be particularly effective for blood glucose lowering without displaying commensurate increases in appetite reduction or nausea is that their effects may be experienced predominantly by the pancreatic beta cell rather than in anorectic neurons. Whilst GLP‐1 receptor in vagal afferent neurons is important for the satiating effects of physiological GLP‐1 released from the gut (Krieger et al., 2015), a study with brain‐specific GLP‐1 receptor knockout mice demonstrated that the anorectic effects of pharmacological GLP‐1 agonists originate mainly in the CNS (Sisley et al., 2014). Possible reasons why biased or partial GLP‐1 agonists might exert tissue‐specific actions are discussed below.

#### 4.2.1 Access to the CNS

The GLP‐1 receptor is expressed in a number of different brain regions (Cork et al., 2015), not all of which are necessarily accessible to peripherally administered GLP‐1 agonists; some areas may instead primarily respond to brain‐derived GLP‐1 from nearby preproglucagon‐expressing neurons. The passage of GLP‐1 agonists across the blood–brain barrier is incompletely understood, but it has been suggested that it might involve GLP‐1 receptor‐dependent ligand uptake and transcytosis across tanycytes (Gabery et al., 2020), the choroid plexus (Botfield et al., 2017), or endothelial cells (Fu et al., 2020), that act as conduits between the peripheral circulation and the brain. This raises the question as to whether biased (or partial) GLP‐1 agonists, which often show reduced GLP‐1 receptor endocytosis, may consequently experience diminished or delayed entry into the CNS, thereby limiting their ability to realise the full anorectic response predicted by their advantageous pharmacology. This possibility has not been fully explored, although it is notable that the GLP‐1 antagonist exendin(9–39), which does not induce GLP‐1 receptor internalisation, can also enter the brain after peripheral administration (Ast et al., 2020). GLP‐1 receptor endocytosis‐dependent transcytosis would also require a mechanism by which GLP‐1 agonists can escape from late endosomal intralumenal vesicles, to which the activated GLP‐1 receptor is usually trafficked (Jones, Buenaventura, et al., 2018).

#### 4.2.2 Tissue bias

Operational models theoretically quantify biased agonism in a system‐independent way, that is, the same degree of bias should be present in different tissues (Kenakin et al., 2012). However, this is not necessarily the case if signalling intermediates are subject to diverse upstream regulatory processes that vary between cell types or are themselves controlled by agonist stimulation, for example Ca^2+^ responses in excitable versus non‐excitable cells or where a readout is dependent on several Gα subtypes with cell‐specific differences in expression. Moreover, even if the bias profile is preserved, tissue‐specific manifestations of biased signalling are clearly dependent on how upstream pathway activation is coupled to downstream responses. Here, system sensitivity is a critical parameter governing the physiological effects of biased agonists, due to, for example, differences in expression levels of the target receptor, upstream and downstream effectors, and signal amplification processes. Thus, a low efficacy agonist may stimulate an observable response in a highly sensitive tissue but not in a tissue with low inherent sensitivity (Kenakin, 1984, 2018).

**FIGURE 2:**
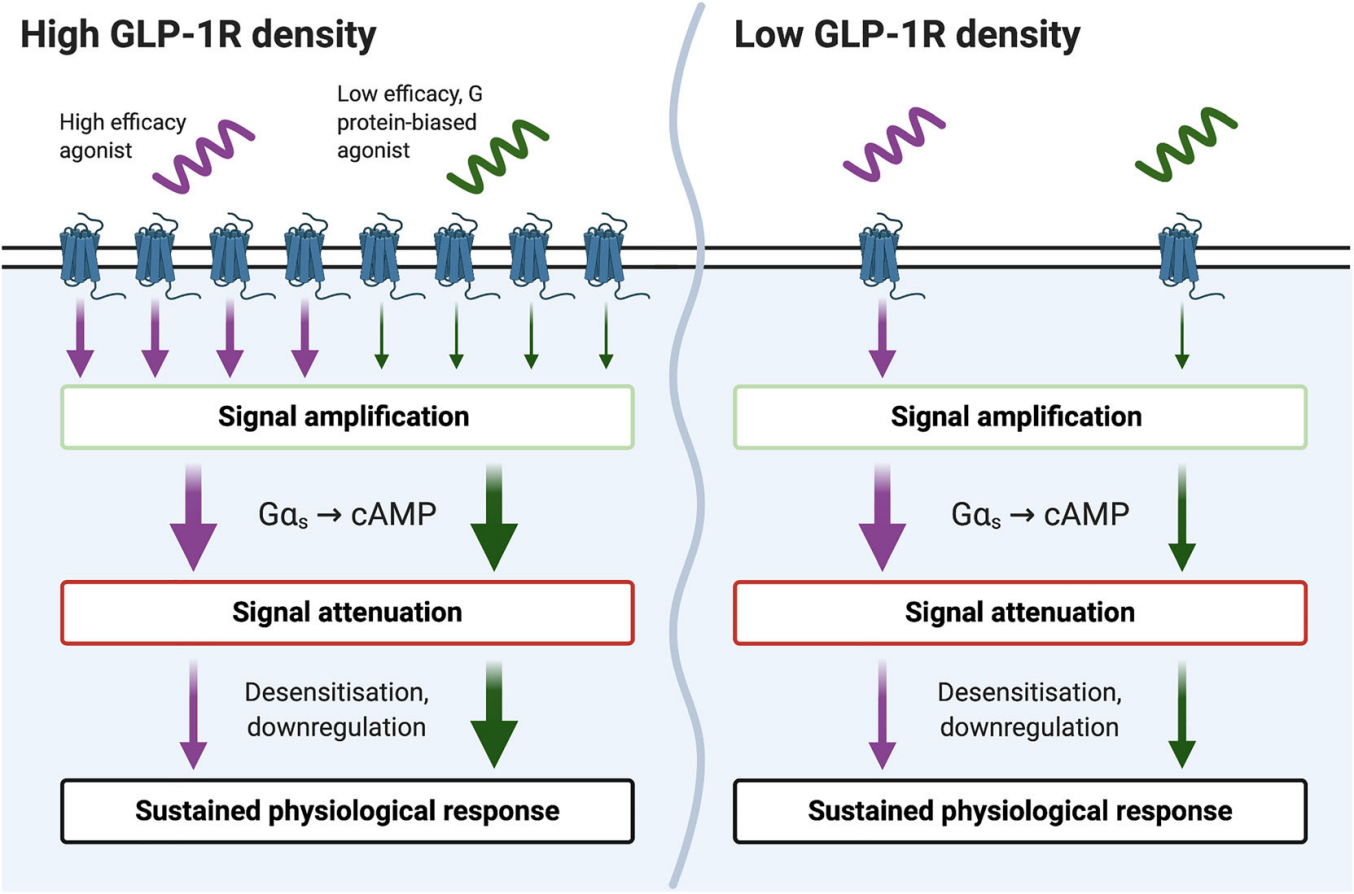
The predicted impact of GLP‐1 receptor (GLP‐1R) density on physiological responses to low efficacy biased GLP‐1 receptor agonists. The G protein‐biased GLP‐1 agonist shows reduced efficacy for both G protein coupling and desensitisation events such as β‐arrestin recruitment. The size of the arrows represents the amplitude of the signal after passing through different stages of the signal transduction pathway. With adequate GLP‐1 receptor reserve, signal amplification by adenylate cyclase allows this low efficacy agonist to generate a full cAMP response. Reduced desensitisation then ensures that the resultant physiological effects (e.g. insulin secretion) persist for longer than with the high efficacy agonist. In contrast, when GLP‐1 receptor density is limited, the low efficacy G protein biased agonist fails to generate a full cAMP response. It still “benefits” from reduced desensitisation, so its physiological effect can match that of the high efficacy agonist. Figure created with BioRender.com

These theoretical considerations may well be relevant to GLP‐1 receptor biased agonism due to the wide variation in GLP‐1 receptor expression levels between target tissues. In particular, GLP‐1 receptor is highly expressed in beta cells but at much lower levels in neurons and other tissues (Richards et al., 2014). Many G protein‐biased GLP‐1 agonists, such as exendin‐phe1 and derivatives, are in fact partial agonists for G protein coupling, with mini‐G_s_ recruitment efficacy reduced by as much as 80% compared to full agonist GLP‐1 receptor ligands (Lucey et al., 2020). These peptides still favour G protein responses on efficacy grounds, as β‐arrestin‐2 recruitment is reduced to an even greater extent in matched assays. In spite of the substantially reduced coupling to Gα_s_, exendin‐phe1 acts as a full agonist for acute cAMP production in pancreatic beta cells as well as heterologous cell lines (Jones, Bloom, et al., 2018), presumably due to the availability of spare receptors and signal amplification. However, it is not clear whether the same applies in anorectic neurons, where GLP‐1 receptor expression is much lower and thus maximal receptor occupancy by exendin‐phe1 may, theoretically, be insufficient to yield a full cAMP response. This principle has been demonstrated for the small molecule GLP‐1 agonist LY3502970, which shows partial agonist activity for cAMP in low‐expressing cell lines but is a full agonist when GLP‐1 receptor expression is high (Kawai et al., 2020). Therefore, lower efficacy G protein‐biased GLP‐1 agonists may achieve a high amplitude response in “sensitive” cells after amplification, which then becomes an extended duration, high amplitude response through avoidance of target desensitisation; in contrast, in a less sensitive cell type, a full amplitude downstream response might never be achieved (Figure 2). This provides a framework through which exaggerated effects on insulin release can occur without increased nausea, but this remains to be confirmed experimentally. It should be highlighted that balanced partial agonists with globally reduced efficacy for coupling to both G proteins and β‐arrestins should theoretically behave similarly, that is, formally confirmed biased agonism is not a requirement. Of course, several other factors beyond receptor density may influence the relative signalling efficacy of different GLP‐1 agonists, such as the presence of other membrane proteins and the lipid microenvironment, tissue‐specific expression of adenylate cyclase isoforms and propagation of upstream signalling to downstream responses. Studies in primary GLP‐1 receptor‐expressing cells are needed to conclusively establish whether the efficacy rank order for biased GLP‐1 agonists differs between tissues.

## 5 CONCLUSIONS

GLP‐1 receptor activation leads to diverse intracellular signalling events linked to a wide variety of cellular and physiological responses. Combined with the availability of high throughput assays to measure these pathways, the exploration of GLP‐1 receptor biased agonism over the past decade has been rapid. Nevertheless, the field is hampered by incomplete understanding of how these pathways are linked to specific downstream effects in native GLP‐1 receptor‐expressing tissues. At this stage, it appears more likely that physiological responses represent the convergence of multiple activated signalling pathways, rather than being individually attributable to unique signalling events. In particular, the role of β‐arrestins in initiating G protein‐independent GLP‐1 receptor signalling versus terminating G protein signalling remains an open question. Although increasing evidence from biased GLP‐1 agonist studies has not identified the loss of any particular pharmacodynamic feature that can be clearly linked to diminished β‐arrestin‐mediated signalling, pointing instead to a core role for avoidance of β‐arrestin‐mediated desensitisation in amplifying G protein‐dependent therapeutic effects such as insulin release. Nevertheless, further studies in mice with targeted deletion of β‐arrestin‐1 or ‐2 are required to clarify whether β‐arrestins are indeed responsible for these effects.

Caution is also required when extrapolating results from biased GLP‐1 agonists tested in rodent models. Allometrically scaled dosing in mice typically achieves higher circulating drug concentrations than is possible in humans. As β‐arrestin recruitment to the GLP‐1 receptor is typically detected at considerably higher ligand concentrations than required for G protein‐dependent responses such as cAMP, it is possible that manifestations of biased GLP‐1 receptor agonism will be less apparent at clinically appropriate doses. Nevertheless, the beneficial effects of P5 over exendin‐4 were most obvious at the lowest concentration tested. Moreover, biased GLP‐1 receptor agonism is touted as one possible explanation for the impressive metabolic effects of tirzepatide, although its activity at the GIP receptor cannot be discounted. A dedicated human study comparing oppositely biased GLP‐1 receptor mono‐agonists is needed to address this question directly. Finally, promising results from biased GLP‐1 agonist studies raise the question of whether it will be possible to harness similar phenomena with other metabolically relevant class B GPCRs, including GIP and glucagon receptors. This question is beginning to be addressed (Darbalaei et al., 2020; Jones et al., 2020; Killion et al., 2020; Yuliantie et al., 2020) and may be a route to enhance metabolic disease treatment through simultaneous targeting of multiple GPCRs in a pathway‐selective manner.

### 5.1 Nomenclature of targets and ligands

Key protein targets and ligands in this article are hyperlinked to corresponding entries in the IUPHAR/BPS Guide to PHARMACOLOGY http://www.guidetopharmacology.org and are permanently archived in the Concise Guide to PHARMACOLOGY 2019/20 (Alexander, Christopoulos, et al., 2019; Alexander, Fabbro, et al., 2019).

## AUTHOR CONTRIBUTIONS

B.J. wrote the article.

## CONFLICT OF INTEREST

B.J. has received grant funding from Sun Pharmaceuticals.

## Data Availability

Data sharing is not applicable as no new data were generated.
